# The Middle Fossa Approach for the Removal of a Trochlear Schwannoma

**DOI:** 10.1155/2014/672314

**Published:** 2014-03-12

**Authors:** Andrew B. Boucher, L. Madison Michael

**Affiliations:** ^1^College of Medicine, University of Tennessee Health Science Center, 910 Madison, Suite 1002, Memphis, TN 38163, USA; ^2^Department of Neurosurgery, University of Tennessee Health Science Center, 847 Monroe Avenue, Suite 427, Memphis, TN 38163, USA; ^3^Semmes-Murphey Neurologic & Spine Institute, 6325 Humphreys Boulevard, Memphis, TN 38120, USA

## Abstract

*Objectives*. Schwannomas originating from the trochlear nerve are extremely rare; only 30 cases have been reported in the literature. Many operative approaches have been utilized for lesion resection, but the advantages of the anterior transpetrosal approach are numerous and include excellent exposure, minimal extradural retraction of the temporal lobe, and minimal cerebrospinal fluid leaks. We report the second case of a trochlear schwannoma resected via the anterior transpetrosal approach. *Setting*. A 64-year-old male presented with 3-month history of diplopia and headaches. On physical examination, he was found to have a right fourth nerve palsy. Brain magnetic resonance imaging revealed a mass within the right ambient cistern compressing the adjacent midbrain. A right-sided anterior transpetrosal approach was used—which confirmed that the trochlear nerve entered the mass—to achieve gross total resection. Pathological examination confirmed diagnosis of schwannoma. The patient was discharged on postoperative day 3. He experienced a persistent fourth nerve palsy postoperatively with an otherwise normal neurological examination. Follow-up imaging confirmed complete removal of the tumor. *Conclusion*. The anterior transpetrosal approach is an excellent approach for removal of trochlear schwannomas involving the cisternal course of the trochlear nerve. It affords complete visualization of this anatomical region while introducing minimal morbidity.

## 1. Introduction

Schwannomas originating from cranial nerves account for 8% of intracranial neoplasms. The vast majority of these tumors arise from the sensory cranial nerves, most commonly, the vestibular or trigeminal nerve [1–3]. Schwannomas that originate from purely motor cranial nerves in patients without neurofibromatosis are uncommon. Of these, trochlear nerve schwannomas are exceedingly rare; to date, there have been only 30 reported cases of surgically confirmed trochlear nerve schwannomas [2–28].

Tumors arising from the trochlear nerve can present a surgical challenge because they often involve the cisternal region near the brainstem [16]. The most common approach used to resect these tumors has been the subtemporal transtentorial approach [16]. But the disadvantages of intradural approaches include retraction injury and cerebrospinal fluid (CSF) leak. The extradural anterior transpetrosal approach is ideal for trochlear schwannomas involving the cisternal portion. It affords excellent visualization of the epicenter of the lesion. Progressive enfolding of the tumor capsule into the surgical opening allows for a radical resection. Other benefits of the anterior transpetrosal approach include minimal temporal lobe retraction, avoidance of vital structures, and minimal CSF leakage. One previously published case employed the anterior transpetrosal approach for resection of a trochlear schwannoma. This case is the second one reported in the literature in which an extradural anterior petrosal approach was used in the resection of a trochlear schwannoma.

## 2. Case Report

### 2.1. History and Examination

A 64-year-old white male with an unremarkable past medical history presented with acute-onset diplopia. His symptoms began 2 months prior to his initial neurosurgical evaluation. There was no obvious history of trauma. The patient reported that his double vision was exacerbated by looking down and was improved when tilting his head to the left. He also was experiencing significant right frontal headaches that worsened throughout the day. On physical examination, he was found to have diplopia on downward gaze to the right, which suggested a right-sided trochlear nerve palsy with no other focal neurological deficits. The remainder of his neurological exam was unremarkable.

### 2.2. Imaging

**Figure 1:**
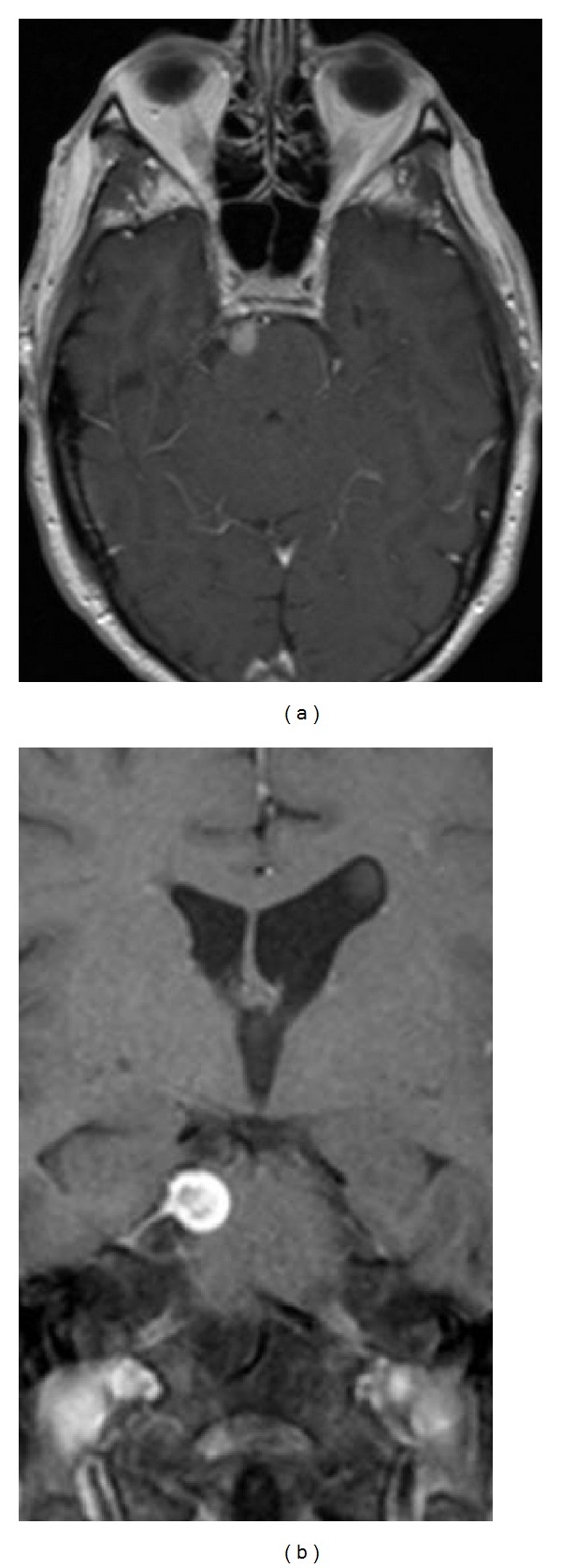
Preoperative MRI brain with contrast axial (a) and coronal T1-weighted (b) images reveals a well-circumscribed, heterogeneously enhancing lesion adjacent to the right side of the upper pons.

Magnetic resonance imaging (MRI) of the brain with and without contrast revealed a well-circumscribed, heterogeneously enhancing space-occupying lesion in the right ambient cistern. The mass measured 9 mm × 7 mm × 11 mm and compressed the adjacent anterior aspect of the upper pons. It appeared to have a broad base with a thin enhancing tail extending inferolaterally along the meninges (Figures 1(a) and 1(b)) [1–3].

### 2.3. Operation

The patient was transferred to the operative table and placed in the lateral decubitus position. A lumbar drain was placed at this time. His head was placed in a Mayfield 3-point fixation system, which was aligned to maximally expose the right temporal region. The operative site was prepped and draped in the usual sterile fashion. A curvilinear incision was made overlying the right temporal region that began approximately 1.5 cm anterior to the external auditory canal and was carried superiorly to the superior temporal line. It was then carried posteriorly approximately 5 cm. The skin flap was rotated inferiorly and posteriorly and affixed into place with hooks. The temporalis muscle was mobilized anteriorly. This exposed the right temporal region. An electric drill was taken, and a right temporal craniotomy was elevated and the microscope was moved into the field.

Approximately 20 mL of CSF was removed from the lumbar drain at this time to promote brain relaxation. An extradural subtemporal dissection ensued. Dissection continued medially and the greater superficial petrosal nerve was identified. Dissection continued medially until Meckel's cave was identified extradurally. Anteriorly, the middle meningeal artery was seen coursing through the foramen spinosum and posteriorly was the superior petrosal sinus.

**Figure 2:**
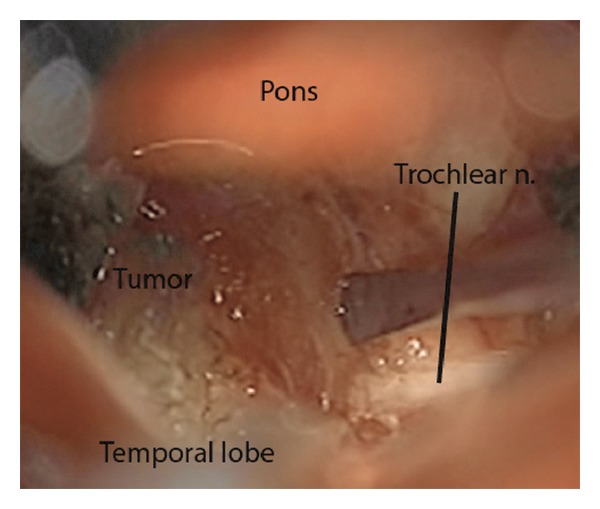
The trochlear nerve entering directly into the visualized tumor.

A middle fossa retractor was inserted and extradural elevation of the right temporal lobe was performed. Careful attention was paid to avoid excessive retraction of the temporal lobe. An anterior petrosectomy was performed as originally described by Kawase et al. [29, 30]. The superior petrosal sinus was cauterized laterally. An incision was made beginning along the temporal dura and carried through the superior petrosal sinus and into the posterior fossa. This allowed identification of the 5th nerve at the nerve root entry zone. The tentorium was cut medially, and the dura overlying Meckel's cave was removed resulting in excellent visualization of the tentorial incisura region. A large mass was clearly identified at this time; the trochlear nerve appeared to enter directly into the lesion (Figure 2). The capsule was cauterized and incised. Multiple specimens were sent for pathological analysis, and the pathology of the specimens was consistent with a schwannoma. The trochlear nerve was now cut proximally to allow further mobilization of the mass. Following intratumoral debulking, progressive mobilization of the capsule resulted in a gross total resection. The retractor was removed, the wound was closed in a multilayer fashion, and a dressing was applied.

### 2.4. Postoperative Course

**Figure 3:**
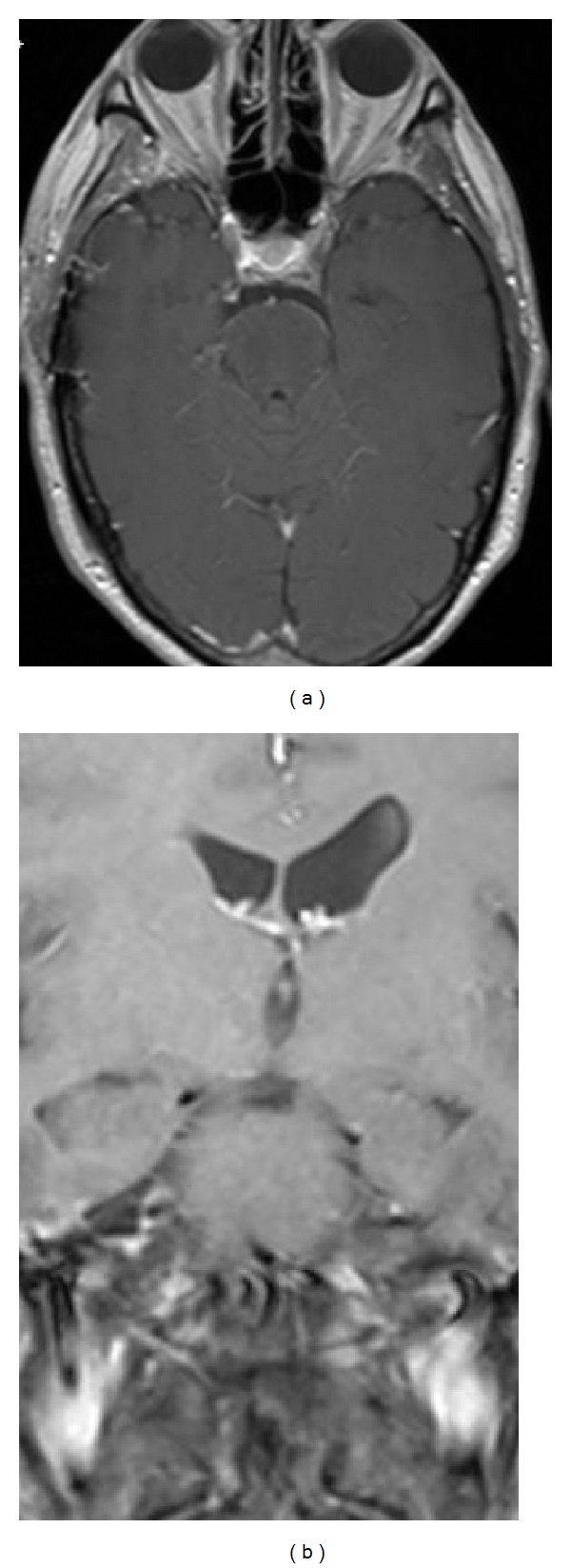
Postoperative MRI brain with contrast axial (a) and coronal (b) T1-weighted images reveals complete resection of the trochlear schwannoma.

The patient had an uneventful postoperative course and was discharged to home on postoperative day 3. As expected, the patient continued to experience diplopia with downward gaze. At 1-month followup, the patient had recovered well from the surgery with no complaints. The right trochlear nerve palsy persisted as anticipated. Followup MRI of the brain performed 6 months postoperatively revealed no evidence of residual disease (Figures 3(a) and 3(b)).

## 3. Discussion

Nonvestibular cranial nerve schwannomas are extremely uncommon. Of these, trigeminal schwannomas are the most common but only account for up to 8% of intracranial schwannomas [31]. Trochlear schwannomas are extremely rare as this case represents just the 32nd reported case that was surgically confirmed (Table 1). Trochlear schwannomas arise mostly in middle-aged patients with an average age of 46 years old, although they have been described in patients as young as 16 years old (Table 1). Trochlear schwannomas may have a variety of presentations ranging from isolated trochlear nerve palsy—as in this case—to hemiparesis, cerebellar signs, or other cranial nerve deficits [16]. Trochlear nerve palsy has only been seen in about half of the surgically confirmed trochlear schwannomas [3, 16]. It has been proposed that due to the cisternal length of the fourth nerve, the tumor may displace and twist the nerve fibers instead of destroying them, which could account for the absence or later presentation of trochlear paralysis in these cases [25]. In our present case, the diplopia was the only presenting symptom, other than headache, suggesting that trochlear schwannomas may present with an isolated trochlear palsy. As a general rule, resection of the trochlear schwannoma entails sacrificing the trochlear nerve, which results in a permanent fourth nerve palsy. However, there has been one recent exception reported in which partial resection resulted in improvement of diplopia [5].

The approach for gross resection of trochlear schwannomas should be based on the size and location of the tumor. The size can vary, but symptomatic tumors that were previously resected ranged from 10 to 45 mm [3]. The vast majority of these tumors arise from the cisternal portion of the fourth nerve (Table 1). This location can pose a challenge due to the morbidities associated with the different surgical approaches needed to reach this region. In the past, the subtemporal transtentorial approach with minor variations has been the most common approach used to resect these tumors (Table 1). Other intradural approaches—including pterional, suboccipital, and presigmoid transpetrosal—have been employed to resect trochlear schwannomas. However, these intradural approaches have increased risk of morbidity due to the greater temporal lobe retraction and increased risk of CSF leaks as compared with the extradural approach used in this case.

We utilized the extended middle fossa approach, also known as the anterior transpetrosal approach, in the gross total resection of the trochlear schwannoma. Kawase et al. first described this approach in 1985 for basilar artery aneurysms and later for the resection of sphenopetroclival meningiomas in 1991 [29, 30]. This approach offers many theoretical advantages over the other options used for resection of trochlear schwannomas, such as great exposure to the petroclival region, which allows for complete resection. With their typical soft consistency, trochlear schwannomas may be easily resected from the cisternal region, which is optimally visualized via the anterior transpetrosal approach.

While the subtemporal transtentorial approach also allows for adequate visualization of the surgical field, this approach potentially involves more temporal lobe retraction than the middle fossa approach. The increased traction increases the likelihood of morbidity due to damage of the temporal lobe. Another potential disadvantage of this approach is harm to the vein of Labbe with resultant venous infarction. The lateral suboccipital approach has also been used successfully, but this approach has disadvantages of inadequate exposure if there are significant parasellar portions of the tumor as well as increased risk of damage to the cranial nerves that surround the access route [29]. The other major advantage of the middle fossa approach is the avoidance of CSF leaks as the dissection is extradural. While CSF leaks have still been reported to occur in some cases [32], the likelihood is greatly decreased as compared to intradural approaches.

The main disadvantages of the middle fossa approach are possible inadequate exposure, if the petrosal bone resection is insufficient, possible unintentional entry into the middle ear, and risk to the geniculate ganglion, if there is excessive traction on the greater superficial petrosal nerve [29, 32]. These risks are minimal with careful dissection and adequate knowledge of the anatomy of the middle fossa.

## 4. Conclusion

The anterior transpetrosal approach is an excellent approach for the resection of trochlear nerve schwannomas. It was used successfully in this patient to achieve gross total resection of this uncommon tumor. The patient retained his isolated right trochlear nerve palsy with no added morbidity. The anterior transpetrosal approach has advantages over other techniques for removal of these rare schwannomas. This approach offers optimal visualization of the petroclival region while introducing minimal morbidity and should be considered as a surgical corridor for removal of trochlear schwannomas with involvement of the ambient cistern.

## Figures and Tables

**Table 1:** Prior Surgically-Defined Trochlear Schwannomas.

Author	Year	Age/Sex	Location	Approach
King [15]	1976	55/F	Ambient Cistern	Subtemporal transtentorial
Boggan et al. [7]	1979	32/F	Ambient Cistern	Subtemporal transtentorial
Leunda et al. [2]	1982	54/M	Ambient Cistern	Subtemporal transtentorial
Leunda et al. [2]	1982	16/F	Cisternocavernous	Subtemporal transtentorial
Yamamoto et al. [27]	1987	37/F	Ambient Cistern	Subtemporal transtentorial
Garen et al. [11]	1987	18/F	Ambient Cistern	Subtemporal transtentorial
Tokuriki et al. [24]	1988	43/M	Ambient Cistern	Subtemporal transtentorial
Maurice-Williams [18]	1989	56/M	Ambient Cistern	Suboccipital, CP angle approach
Samii et al. [20]	1989	53/F	Ambient Cistern	Pterional
Celli et al. [8]	1992	51/M	Ambient Cistern	Subtemporal transtentorial
Jackowski et al. [14]	1994	26/F	Ambient Cistern	Transtemporal with partial division of tentorium
Abe et al. [4]	1994	60/M	Ambient Cistern	Lateral Suboccipital
Abe et al. [4]	1994	57/M	Ambient Cistern	Subtemporal transtentorial
Dolenc and Coscia [9]	1996	68/M	Ambient Cistern	Lateral suboccipital
Beppu et al. [6]	1997	66/M	Ambient Cistern	Lateral suboccipital
Santoreneos et al. [21]	1997	35/F	Ambient Cistern	Subtemporal with partial division of tentorium
Nadkarni and Goel [19]	1999	48/F	Ambient Cistern	Subtemporal transtentorial
Matsui et al. [17]	2002	61/M	Ambient Cistern	Presigmoid transpetrosal
Veshchev et al. [26]	2002	26/F	Cavernous Sinus	Pterional
Türe et al. [25]	2002	31/M	Ambient Cistern	Infratentorial, lateral supracerebellar
Shenouda et al. [22]	2002	49/M	Cisternocavernous	Presigmoid combined transpetrosal
Du et al. [10]	2003	17/F	Ambient Cistern	Orbitozygomatic pterional
Shenoy and Raja [23]	2004	54/F	Ambient Cistern	Subtemporal transtentorial
Ohba et al. [3]	2006	48/M	Ambient Cistern	Anterior transpetrosal
Gerganov et al. [12]	2007	52/F	Ambient Cistern	Retrosigmoid
Grigorian and Korobova [13]	2008	47/F	Ambient Cistern	Retromastoidal
Grigorian and Korobova [13]	2008	44/F	Ambient Cistern	Paramedian subtentorial supracerebellar
Kohama et al. [16]	2009	47/F	Ambient Cistern	Posterior transpetrosal
Bartalena et al. [5]	2010	50/F	Ambient Cistern	Subtemporal transtentorial
Younes et al. [28]	2012	65/F	Ambient Cistern	Pterional
Boucher and Michael (current study)	2013	64/M	Ambient Cistern	Anterior transpetrosal
